# Comparative genome analysis of 52 fish species suggests differential associations of repetitive elements with their living aquatic environments

**DOI:** 10.1186/s12864-018-4516-1

**Published:** 2018-02-13

**Authors:** Zihao Yuan, Shikai Liu, Tao Zhou, Changxu Tian, Lisui Bao, Rex Dunham, Zhanjiang Liu

**Affiliations:** 1 The Fish Molecular Genetics and Biotechnology Laboratory, Aquatic Genomics Unit, School of Fisheries, Aquaculture and Aquatic Sciences and Program of Cell and Molecular Biosciences, Auburn University, Auburn, AL 36849 USA; 2 Department of Biology, College of Art and Sciences, Syracuse University, Syracuse, NY 13244 USA

**Keywords:** Fish, Evolution, Repeat, Transposon, Microsatellite, Habitat

## Abstract

**Background:**

Repetitive elements make up significant proportions of genomes. However, their roles in evolution remain largely unknown. To provide insights into the roles of repetitive elements in fish genomes, we conducted a comparative analysis of repetitive elements of 52 fish species in 22 orders in relation to their living aquatic environments.

**Results:**

The proportions of repetitive elements in various genomes were found to be positively correlated with genome sizes, with a few exceptions. More importantly, there appeared to be specific enrichment between some repetitive element categories with species habitat. Specifically, class II transposons appear to be more abundant in freshwater bony fish than in marine bony fish when phylogenetic relationship is not considered. In contrast, marine bony fish harbor more tandem repeats than freshwater species. In addition, class I transposons appear to be more abundant in primitive species such as cartilaginous fish and lamprey than in bony fish.

**Conclusions:**

The enriched association of specific categories of repetitive elements with fish habitats suggests the importance of repetitive elements in genome evolution and their potential roles in fish adaptation to their living environments. However, due to the restriction of the limited sequenced species, further analysis needs to be done to alleviate the phylogenetic biases.

**Electronic supplementary material:**

The online version of this article (10.1186/s12864-018-4516-1) contains supplementary material, which is available to authorized users.

## Background

The majority of eukaryotic genomes contain a large proportion of repetitive elements. Based on their arrangements in the genome, repetitive elements can be divided into two major categories: the transposable elements or transposons and the tandem repeats. Transposons can be divided into RNA-mediated class I transposons, which include transposons with long terminal repeats (LTRs), long interspersed nuclear elements (LINEs), and short interspersed nuclear elements (SINEs); and RNA-independent class II DNA transposons. Tandem repeats are copies of DNA repeats located adjacent to one other [1–3]. Tandem repeats themselves can be dispersed across the whole genome such as the case of microsatellites, and they can be clustered in the highly repetitive genome regions such as centromeric, telomeric and subtelomeric regions [4, 5].

Although repetitive elements were considered to be junk DNA [6], recent studies suggested that they are functional in regulating gene expression and contribute to genome evolution [7–11]. Transposons are considered to be drivers of genetic diversification because of their ability to co-opt into genetic processes such as restructuring the chromosomes or providing genetic material on which natural selection can act on [12–14], and thus can be the major reason for species difference in genome size [15–17]. Similarly, expansion or contraction of tandem repeats can also affect genome size [18–20], and consequently affect recombination, gene expression, and conversion and chromosomal organization [21–26].

Fish comprise a large and highly diverse group of vertebrates inhabiting a wide range of different aquatic environments [27]. Sequenced fish genomes vary in size from 342 Mb of *Tetraodon nigroviridis* to 2967 Mb of *Salmo salar*. Some studies have been conducted on the diversity of repetitive elements in fish [28–30], but systematic comparative studies have been hindered by the lack of whole genome sequences from a large number of species. Recent availability of a large number of fish genome sequences made it possible to determine the repetitive element profiles of fish species from a broad taxonomic spectrum. In this study, we annotated the repetitive elements of 52 fish genomes from 22 orders, and determined their distribution in relationship with environmental adaptations. Here, we observed the correlation between high numbers of DNA transposons, especially the Tc1 transposons, with freshwater bony fish, high level of microsatellites with marine bony fish, and high numbers of class I transposons with cartilaginous fish and lamprey. Based on the phylogeny tree, the effects of phylogeny on the differences between freshwater or marine bony fish were evaluated with the phylogenetically independent contrasts (PIC).

## Results

### Contents of repetitive elements in various fish genomes

**Fig. 1:**
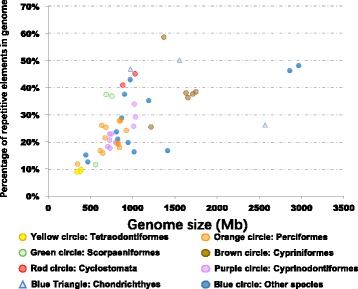
Correlation between genome sizes and contents of repetitive elements. Genome sizes against the percentages of repetitive elements to the whole genome are plotted for 52 species of species for which genome sequences are available. The major orders are plotted in different colors and shapes: Yellow circle: Tetraodontiformes; Orange circle: Perciformes circle; Green circle: Scorpaeniformes; Brown circle: Cypriniformes; Red circle: Cyclostomata; Purple circle: Cyprinodontiformes; Blue triangle: Chondrichthyes; Blue circle: Other species

A total of 128 categories of repetitive elements are identified from the 52 fish species (Additional file 1: Table S1). We found overall positive correlation between contents of repetitive elements in fish and their genome sizes. This correlation, was still significant when implementing phylogenetically independent contrasts (Fig. 1, PIC *p*-value: 1.88e-03, Pearson correlation r = 0.6, *p*-value = 1.45e-06). However, several exceptions existed. For instance, the whale shark genome is 2.57 Gb, but contains only 26.2% of repetitive elements; in contrast, the mid-sized zebrafish genome is ~ 1.5 Gb in size, but contains over 58% of repetitive elements.

### Differential associations of repetitive elements across species

**Fig. 2:**
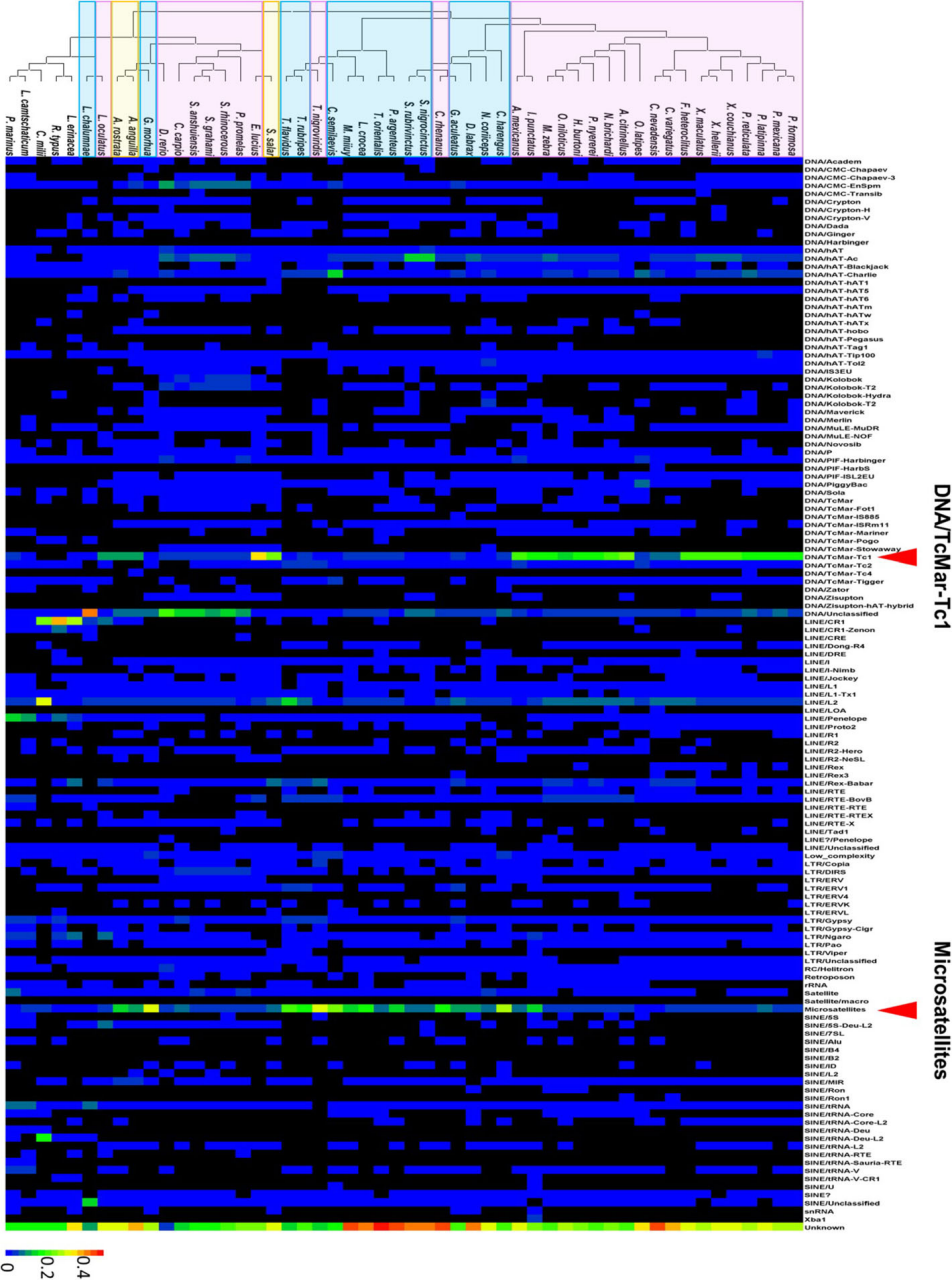
Classification and distribution of 128 repetitive elements in 52 species. The total number of each category of repeats to the all repeats are displayed in columns while different species are displayed in rows. The pink shade represents the freshwater living bony fish, the blue represents the marine living bony fish and yellow represents the diadromous species

We investigated the possible association between repetitive elements and aquatic environment. Comparison of diversity and abundance of repetitive elements across the 52 fish genomes revealed significant differences among species (Fig. 2 and Additional file 2: Table S2). Class I transposons are more prevalent in cartilaginous fish and lampreys than bony fish species (Wilcoxon rank test, *p*-value = 1.41e-04). For example, class I transposons represent 76.6% of repetitive elements in elephant shark, but the bony fish genomes are more abundant with class II transposons and tandem repeats.

Of the bony fish genomes, the freshwater bony fish contained a greater proportion of Tc1/mariner transposons than marine species (Fig. 2, Wilcoxon rank test, *p*-value = 8.23e-06). However, the results were not significant when the phylogeny was taken into consideration (PIC p- value: 0.117). In contrast, the marine bony fish contain a greater proportion of microsatellites (PIC p-value: 3.12e-02, Wilcoxon rank test, *p*-value = 3.72e-05) than the freshwater species, independent of the phylogeny. Interestingly, the diadromous species such as *Anguilla rostrata*, *Anguilla anguilla*, and *S. salar* contain high proportions of both the Tc1/mariner transposons and microsatellites (Table 1).

**Table 1:** Proportion of DNA/TcMar-Tc1, microsatellites contents out of all repeats in freshwater, marine and diadromous teleost species

	Species	Order	DNA/TcMar-Tc1	Microsatellites
Freshwater species	*Esox lucius*	Esociformes	35.9%	4.8%
	*Fundulus heteroclitus*	Cyprinodontiformes	22.6%	4.6%
	*Xiphophorus hellerii*	Cyprinodontiformes	22.7%	5.1%
	*Xiphophorus couchianus*	Cyprinodontiformes	22.3%	5.1%
	*Amphilophus citrinellus*	Perciformes	23.0%	6.2%
	*Xiphophorus maculatus*	Cyprinodontiformes	22.0%	6.2%
	*Lepisosteus oculatus*	Lepisosteiformes	10.5%	3.0%
	*Pundamilia nyererei*	Perciformes	18.7%	5.8%
	*Haplochromis burtoni*	Perciformes	19.6%	6.1%
	*Maylandia zebra*	Perciformes	16.8%	5.3%
	*Neolamprologus brichardi*	Perciformes	20.9%	6.7%
	*Cyprinodon nevadensis*	Cyprinodontiformes	7.6%	2.5%
	*Poecilia formosa*	Cyprinodontiformes	19.3%	6.5%
	*Oreochromis niloticus*	Perciformes	15.9%	5.4%
	*Poecilia reticulata*	Cyprinodontiformes	17.8%	6.1%
	*Poecilia mexicana*	Cyprinodontiformes	18.9%	6.7%
	*Astyanax mexicanus*	Characiformes	21.8%	8.0%
	*Poecilia latipinna*	Cyprinodontiformes	19.5%	7.4%
	*Cyprinodon variegatus*	Cyprinodontiformes	8.2%	3.4%
	*Oryzias latipes*	Beloniformes	5.0%	2.6%
	*Ictalurus punctatus*	Siluriformes	19.9%	14.1%
	*Danio rerio*	Cypriniformes	6.1%	5.9%
	*Cyprinus carpio*	Cypriniformes	6.4%	7.1%
	*Sinocyclocheilus grahami*	Cypriniformes	4.7%	5.7%
	*Sinocyclocheilus rhinocerous*	Cypriniformes	3.4%	6.0%
	*Sinocyclocheilus anshuiensis*	Cypriniformes	3.2%	6.2%
	*Pimephales promelas*	Cypriniformes	3.1%	6.7%
	*Cottus rhenanus*	Scorpaeniformes	0.9%	17.8%
	*Tetraodon nigroviridis*	Tetraodontiformes	1.3%	31.1%
Diadromous species	*Salmo salar*	Salmoniformes	23.6%	7.5%
	*Anguilla anguilla*	Anguilliformes	11.9%	11.4%
	*Anguilla rostrata*	Anguilliformes	11.8%	13.9%
Marine species	*Thunnus orientalis*	Perciformes	3.6%	9.3%
	*Pampus argenteus*	Perciformes	5.6%	15.2%
	*Gasterosteus aculeatus*	Gasterosteiformes	4.1%	12.8%
	*Miichthys miiuy*	Perciformes	4.0%	14.7%
	*Notothenia coriiceps*	Perciformes	2.3%	9.5%
	*Dicentrarchus labrax*	Perciformes	2.4%	11.9%
	*Larimichthys crocea*	Perciformes	3.4%	17.7%
	*Takifugu rubripes*	Tetraodontiformes	3.3%	19.9%
	*Sebastes nigrocinctus*	Scorpaeniformes	1.1%	8.9%
	*Cynoglossus semilaevis*	Pleuronectiformes	2.7%	23.1%
	*Takifugu flavidus*	Tetraodontiformes	2.4%	21.8%
	*Sebastes rubrivinctus*	Scorpaeniformes	1.0%	9.1%
	*Clupea harengus*	Clupeiformes	3.0%	29.8%
	*Latimeria chalumnae*	Coelacanthiformes	0.0%	1.7%
	*Gadus morhua*	Gadiformes	0.4%	31.4%

**Fig. 3:**
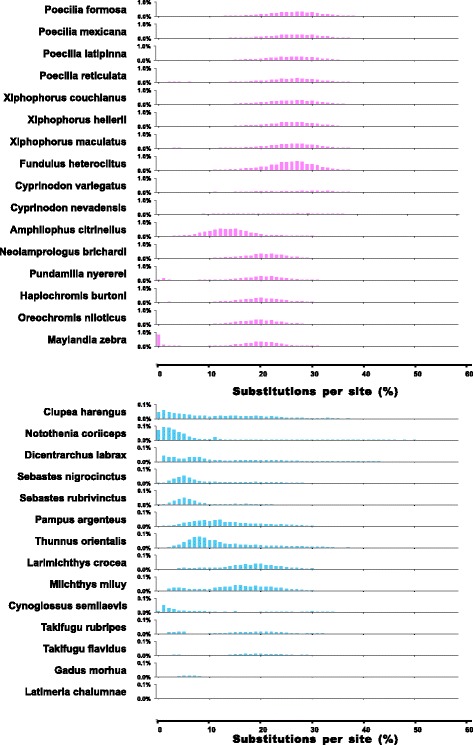
Divergence distribution analysis of DNA/TcMar-Tc1 transposons in the representative fish genomes. The Cyprinodontiformes, Labroidei species (red) and marine bony fish (blue) are displayed. The y-axis represents the percentage of the genome comprised of repeat classes (%) and the x-axis represents the substitution rate from consensus sequences (%). Please note that not all y-axis scales are the same, particularly in marine species which are 10 times smaller

Analysis of the sequence divergence rates suggest that Tc1 transposons have been present in the genomes of freshwater species for much a longer period of time or are more active than in marine species (Fig. 3). The Tc1 transposons in freshwater species are not only more abundant, but also exhibited a higher average K (average number of substitutions per site) (PIC *p*-value: 2.10e-02, Wilcoxon rank test, *p*-value = 5.39e-03) than those in marine species. This is particularly notable in Cyprinodontiformes and Labroidei in Perciformes, where Tc1 transposons appeared to have the strongest activity over a long history, as reflected by the broad distribution and sharp peaks with higher substitution rates per site (Fig. 3). The long history and high transposition activities in freshwater fish accounted, at least in part, for the high proportion of Tc1 transposons in the genomes of freshwater species.

## Discussion

### Accumulation of repetitive elements in fish genomes

In this work, we determined the correlation between the categories and proportions of repetitive elements and the living environments of various fish species. We found that class II transposons appeared to be more abundantly associated with freshwater bony fish than with marine bony fish, when phylogeny was not considered. In contrast, microsatellites are more abundantly associated with marine bony fish than with freshwater bony fish, independent of phylogenetic relationship. In addition, class I transposons are more abundant in primitive species such as cartilaginous fish and lamprey than in bony fish. Such findings suggest that these repetitive elements are related to the adaptability of fish to their living environments, although it is unknown at present if the differential categories and proportions of repetitive elements led to the adaptation to their living environments (the cause) or the living environments led to the accumulation of different repetitive elements (the consequences).

With teleost fish, the genome sizes are greatly affected by the teleost-specific round of whole genome duplication [31–33]. However, whole genome duplication did not dramatically change the proportion of the repetitive elements in the genomes. In contrast, the expansion of repetitive elements may have contributed to the expansion of fish genome sizes as observed in our analysis, fish genome sizes, with exceptions, were found to be well correlated with their contents of repetitive elements. High contents of repetitive elements in the genome can accelerate the generation of novel genes for adaptations, but their overburden can also cause abnormal recombination and splicing, resulting in unstable genomes [34]. Therefore, the content of the repetitive elements cannot grow unlimited with the genome size; it must be limited to certain levels and shaped under specific natural selection by the environment.

It is worthwhile noting that the quality of the genome assembly varied greatly. As one would expect, many of the repetitive elements may have not been assembled into the reference genome sequences, especially with those of lower assembly qualities. This may have affected the assessment of the proportions of the repetitive elements in the genomes. However, most of the genomes sequencing methods are overall similar via next generation sequencing especially Illumina sequencing, thus the systematic biases related to repeat resolution should be small. In addition, if the unassembled repetitive elements are more or less random, the quality of the genome assemblies should not have systematically affected the enrichment of specific categories of repetitive elements with habitats. The total number of genomes used in the study is relatively large (52), the impact of sequence assembly quality should have been minimized.

### Comparison of the repetitive elements among species

The distributions of repetitive elements are significantly associated with various clades during evolution. For example, class I transposons are more prevalent in cartilaginous fish and lampreys than in bony fish species. However, the cartilaginous fish and lamprey lack the class II transposons. Although there were no unifying explanations for this difference, it is speculated that it may be related to the internal fertilization of cartilaginous fish, which may have minimized the exposure of gametes and embryos from horizontal transfer of Class II transposons [30, 35, 36]. Interestingly, active transposable elements in mammals are also RNA transposons. For lamprey, since it is still unclear how it fertilizes and develops in the wild [37, 38], its accumulation of class I transposons deserve further investigation. As class I transposons are involved in various biological processes such as regulation of gene expression [39, 40], the ancient accumulation of class I transposons in cartilaginous fish and lamprey are probably related to their evolutionary adaptations [41]. The contents of class I transposons are low in bony fish; the exact reasons are unknown, but could involve putative mechanisms that counteract the invasiveness of RNAs on their genomes. We realized that a much larger number of bony fish genomes are used in this study than those from cartilaginous fish and lamprey, but this is dictated by the availability of genome sequences. However, if the repetitive elements are more conserved in their categories and proportions of the genome among most closely related species, such bias in the number of genomes used in the analysis should not significantly change the results.

Repetitive elements of most freshwater bony fish are dominated by DNA transposons except *C. rhenanus* and *T. nigroviridis* which contain high levels of microsatellites. Although *T. nigroviridis* is a freshwater species, the vast majority (497 out of 509) of species in Tetraodontidae family are marine species [42–44]. Thus it is likely that *T. nigroviridis* had a marine origin. Similarly, *C. rhenanus* is a freshwater species, but most species of the Cottidae family are marine species [43]. In addition, the biology of *C. rhenanus* is largely unknown [45, 46], and the origin of *C. rhenanus* as a freshwater species remains unexplained.

Uncovering the route of class II transposons expansion is difficult, because they can be transferred both vertically and horizontally [47–49]. However, when phylogenic relationships were not considered, the observed prevalent class II transposon in freshwater species may indicate that the freshwater environments are more favorable for proliferation and spreading of DNA transposons. In addition, as found in other species, the frequent stress such as droughts and floods in the freshwater ecosystem can accelerate transpositions, which facilitate the host adaptions to the environment by generating new genetic variants [50]. Previous studies showed that freshwater ray-finned fish have smaller effective population sizes and larger genome sizes than marine species [51]. Our results lend additional support to the idea that shrinking effective population sizes may have underlined the evolution of more complex genomes [52, 53]. The significance for more prevalence of Tc1 transposon in freshwater species was reduced when accounting for phylogenetic relationship, which indicates the taxa in our data set for analysis are not statistically independent because of shared evolutionary history. However, due to the dictation of the limited and uneven sequenced species available so far, it will inevitably introduce phylogenetic bias into the analysis. For example, a large number of the sequenced fish species belong to the family of Cichlidae (6) or Cyprinidae (6). However, there is only one genome available (*Ictalurus punctatus*) from the order of Siluriformes, which comprise 12% of all fish species [54, 55]. Considering the fact that the phylogenetic independent contrasts analysis is robust to random species sampling [56], thus, further analysis should be conducted with a broader scope with more sequenced fish species, to complement the broader comparative studies.

Although the *Gasterosteus aculeatus* is collected from freshwater, studies indicated that limnetic *G. aculeatus* are formed as a result of marine populations trapped in freshwater recently [57–59]. Thus we still classify the *G. aculeatus* as marine species. Because the population of marine species tend to be more stable than those in freshwater. Besides, the marine teleost species tend to have a higher osmotic pressure of body fluid [60, 61], thus, the high salinity environment may be prone to DNA polymerase slippage while not favorable for proliferation and spreading of transposons, since previous studies indicated that the higher salt concentration might stabilize the hairpin structure during the DNA polymerase slippage [62]. Future research covering a broader scope of sequenced fish linages will address whether passive increases in genome size have in fact been co-opted for the adaptive evolution of complexity in fish as well as other lineages.

## Conclusions

In this study, we investigated the diversity, abundance, and distribution of repetitive elements among 52 fish species in 22 orders. Differential associations of repetitive elements were found from various clades and their living environments. Class I transposons are abundant in lamprey and cartilaginous fish, but less so in bony fish. Tc1/mariner transposons are more abundant in freshwater bony fish than in marine fish when phylogeny was not taken into consideration, while microsatellites are more abundant in marine species than those in freshwater species, independent of phylogeny. The average number of substitutions per sites of Tc1 among bony fish species suggested their longer and more active of expansion in freshwater species than in marine species, suggesting that freshwater environment is more favorable for the proliferations of Tc1 transposons. The analysis of the number of repeats within each microsatellite locus suggested that DNA polymerases are more prone to slippage during replication in marine environments than in freshwater environments. These observations support the notion that repetitive elements have roles for environmental adaptations during evolution. However, whether that is the cause or the consequences requires future studies with more comprehensive sequenced genomes.

## Methods

### Annotation of repetitive elements in fish genome assemblies

The channel catfish genome was assembled by our group [54], the genome sequences of other 51 species were retrieved from NCBI or Ensembl databases [33, 42, 56, 63–89] (Additional file 1: Table S1). The repetitive elements were identified using RepeatModeler 1.0.8 containing RECON [90] and RepeatScout with default parameters [91]. The derived repetitive sequences were searched against Dfam [92] and Repbase [93]. If the sequence is classified as “Unknown”, they were further searched against the NCBI-nt database using blastn 2.2.28 + .

### Phylogenetic analysis

The phylogenetic analysis was based on the cytochrome b [94]. Multiple alignments were conducted by MAFFT [95]. The best substitution model was selected by Prottest 3.2.1 [96]. The phylogenetic tree was constructed using MEGA7 with the maximum likelihood method [97], using JTT with Freqs. (+ F) model, and gaps were removed by partial deletion. The topological stability was evaluated with 1000 bootstraps.

### Divergence distribution of DNA/TcMar-Tc1

The average number of substitutions per sites (K) for each DNA/TcMar-Tc1 fragment was subtotaled. The K was calculated based on the Jukes-Cantor formula: K = − 300/4 × Ln(1-D × 4/300), the D represents the proportion of each DNA/TcMar-Tc1 fragment differ from the consensus sequences [98].

### Statistics and plotting

The statistical analyses for the significance of differences between different groups and the habitats were performed by Wilcoxon rank test function in R language package because the data are not normally distributed [99]. The Pearson correlation analysis in Excel was applied for the correlation between genome size and the content of repetitive elements. Based on the phylogeny tree of the species generated in the previous method, the phylogenetically independent contrasts between the environments and different characters was conducted to evaluate the bias of the phylogeny. The freshwater and sea water was represented by their respective salinities (0.5 for freshwater and 35 for seawater) [100]. The phylogenetically independent contrast test was conducted via the “drop.tip ()” and “pic ()” function in ape package provided by R [101]. The heat map was plotted using the Heml1.0 [102].

## Additional files

Additional file 1: Table S1. Fish genomes used for analysis. (DOCX 33 kb)

Additional file 2: Table S2. Distribution of repetitive elements among species. (XLS 96 kb)

## Abbreviations

DIAG: Data Intensive Academic Grid
ENA: EMBL Nucleotide Sequence Database
K: Average number of substitutions per sites
LINEs: Long interspersed nuclear elements
LTRs: Long terminal repeats
PIC: Phylogenetically independent contrasts
SINEs: Short interspersed nuclear elements

## References

[1] Kubis S, Schmidt T, Heslop-Harrison JSP (1998). Repetitive DNA elements as a major component of plant genomes. Ann Bot.

[2] Tóth G, Gáspári Z, Jurka J (2000). Microsatellites in different eukaryotic genomes: survey and analysis. Genome Res.

[3] Ugarković Ð, Plohl M (2002). Variation in satellite DNA profiles—causes and effects. EMBO J.

[4] Hacch F, Mazrimas J. Fractionation and characterization of satellite DNAs of the kangaroo rat (*Dipodomys Ordii*). Nucleic Acids Res. 1974;1:559–76. 10.1093/nar/1.4.559 PMC343357 10793740

[5] Petitpierre E, Juan C, Pons J, Plohl M, Ugarkovic D. Satellite DNA and constitutive heterochromatin in tenebrionid beetles. In: Kew chromosome conference IV: Royal Botanic Gardens; London. 1995. p. 351-62.

[6] Ohno S (1972). So much “junk” DNA in our genome. Brookhaven symposia in biology.

[7] Meagher TR, Vassiliadis C (2005). Phenotypic impacts of repetitive DNA in flowering plants. New Phytol.

[8] Schmidt AL, Anderson LM (2006). Repetitive DNA elements as mediators of genomic change in response to environmental cues. Biol Rev.

[9] Sun Y-B, Xiong Z-J, Xiang X-Y, Liu S-P, Zhou W-W, Tu X-L, Zhong L, Wang L, Wu D-D, Zhang B-L (2015). Whole-genome sequence of the Tibetan frog Nanorana Parkeri and the comparative evolution of tetrapod genomes. Proc Natl Acad Sci.

[10] Thornburg BG, Gotea V, Makałowski W (2006). Transposable elements as a significant source of transcription regulating signals. Gene.

[11] Wang X, Fang X, Yang P, Jiang X, Jiang F, Zhao D, Li B, Cui F, Wei J, Ma C (2014). The locust genome provides insight into swarm formation and long-distance flight. Nat Commun.

[12] Hurst GD, Werren JH (2001). The role of selfish genetic elements in eukaryotic evolution. Nat Rev Genet..

[13] Kazazian HH (1999). An estimated frequency of endogenous insertional mutations in humans. Nat Genet.

[14] Kazazian HH (2004). Mobile elements: drivers of genome evolution. Science.

[15] Lee S-I, Kim N-S (2014). Transposable elements and genome size variations in plants. Genomics Inform.

[16] SanMiguel P, Tikhonov A, Jin Y-K, Motchoulskaia N (1996). Nested retrotransposons in the intergenic regions of the maize genome. Science.

[17] Wicker T, Sabot F, Hua-Van A, Bennetzen JL, Capy P, Chalhoub B, Flavell A, Leroy P, Morgante M, Panaud O (2007). A unified classification system for eukaryotic transposable elements. Nat Rev Genet.

[18] Charlesworth B, Sniegowski P, Stephan W (1994). The evolutionary dynamics of repetitive DNA in eukaryotes. Nature.

[19] Lindahl T (1994). DNA repair: DNA surveillance defect in cancer cells. Curr Biol.

[20] Strand M, Prolla TA, Liskay RM, Petes TD (1993). Destabilization of tracts of simple repetitive DNA in yeast by mutations affecting DNA mismatch repair. Nature.

[21] Balaresque P, King TE, Parkin EJ, Heyer E, Carvalho-Silva D, Kraaijenbrink T, Knijff P, Tyler-Smith C, Jobling MA (2014). Gene conversion violates the stepwise mutation model for microsatellites in Y-chromosomal palindromic repeats. Hum Mutat.

[22] Hancock JM (1996). Simple sequences and the expanding genome. BioEssays.

[23] Martin P, Makepeace K, Hill SA, Hood DW, Moxon ER (2005). Microsatellite instability regulates transcription factor binding and gene expression. Proc Natl Acad Sci.

[24] Moxon ER, Rainey PB, Nowak MA, Lenski RE (1994). Adaptive evolution of highly mutable loci in pathogenic bacteria. Curr Biol.

[25] Pardue M, Lowenhaupt K, Rich A, Nordheim A (1987). (dC-dA) n.(dG-dT) n sequences have evolutionarily conserved chromosomal locations in drosophila with implications for roles in chromosome structure and function. EMBO J.

[26] Richard GF, Pâques F (2000). Mini-and microsatellite expansions: the recombination connection. EMBO Rep.

[27] Volff J (2005). Genome evolution and biodiversity in teleost fish. Heredity.

[28] Chalopin D, Naville M, Plard F, Galiana D, Volff J-N (2015). Comparative analysis of transposable elements highlights mobilome diversity and evolution in vertebrates. Genome Biol Evol..

[29] Chalopin D, Volff J-N, Galiana D, Anderson JL, Schartl M (2015). Transposable elements and early evolution of sex chromosomes in fish. Chromosom Res.

[30] Gao B, Shen D, Xue S, Chen C, Cui H, Song C (2016). The contribution of transposable elements to size variations between four teleost genomes. Mob DNA.

[31] Allendorf FW, Thorgaard GH. Tetraploidy and the evolution of salmonid fishes. In: Evolutionary genetics of fishes: Springer;Boston.1984. p. 1-53.

[32] Meyer A, Van de Peer Y (2005). From 2R to 3R: evidence for a fish-specific genome duplication (FSGD). BioEssays.

[33] Xu P, Zhang X, Wang X, Li J, Liu G, Kuang Y, Xu J, Zheng X, Ren L, Wang G (2014). Genome sequence and genetic diversity of the common carp, Cyprinus Carpio. Nat Genet.

[34] Jiang H (1997). The distribution trends in simple repetitive stretches of DNA. Chinese J Biochem Mol.

[35] Compagno LJ. Alternative life-history styles of cartilaginous fishes in time and space. Environ Biol Fishes. 1990;28:33-75.

[36] Huang CRL, Burns KH, Boeke JD (2012). Active transposition in genomes. Annu Rev Genet.

[37] Siwicke KA, Seitz AC (2015). Interpreting lamprey attacks on Pacific cod in the eastern Bering Sea. T Am Fish Soc.

[38] Clemens BJ, Binder TR, Docker MF, Moser ML, Sower SA (2010). Similarities, differences, and unknowns in biology and management of three parasitic lampreys of North America. Fisheries.

[39] Brosius J (1999). RNAs from all categories generate retrosequences that may be exapted as novel genes or regulatory elements. Gene.

[40] Brosius J. Genomes were forged by massive bombardments with retroelements and retrosequences. Genetica. 1999;107:209-38. 10952214

[41] Gess RW, Coates MI, Rubidge BS (2006). A lamprey from the Devonian period of South Africa. Nature.

[42] Watson CA, Hill JE, Graves JS, Wood AL, Kilgore KH (2009). Use of a novel induced spawning technique for the first reported captive spawning of Tetraodon Nigroviridis. Mar Genomics.

[43] Nelson J (2006). Fishes of the world 4th edition.

[44] Jaillon O, Aury J-M, Brunet F, Petit J-L, Stange-Thomann N, Mauceli E, Bouneau L, Fischer C, Ozouf-Costaz C, Bernot A (2004). Genome duplication in the teleost fish *Tetraodon nigroviridis* reveals the early vertebrate proto-karyotype. Nature.

[45] Ovidio M, Detaille A, Bontinck C, Philippart J-C (2009). Movement behaviour of the small benthic Rhine sculpin *Cottus rhenanus* (Freyhof, Kottelat & Nolte, 2005) as revealed by radio-telemetry and pit-tagging. Hydrobiologia.

[46] Xiang-Yi L, Nolte AW, Vincx M, Sedlazek F, Konrad K. Genome evolution following admixture in invasive sculpins: Master Thesis, Max-Planck-Institute für Evolutionsbiologie; Plön. 2012.

[47] Abrusán G, Krambeck H-J (2006). Competition may determine the diversity of transposable elements. Theor Popul Biol.

[48] McDonald JF. Evolution and consequences of transposable elements. Curr Opin Genet Dev. 1993;3:855-64. 10.1016/0959-437x(93)90005-a 8118210

[49] Zhang H-H, Feschotte C, Han M-J, Zhang Z (2014). Recurrent horizontal transfers of Chapaev transposons in diverse invertebrate and vertebrate animals. Genome Biol Evol.

[50] Schrader L, Kim JW, Ence D, Zimin A, Klein A, Wyschetzki K, Weichselgartner T, Kemena C, Stökl J, Schultner E (2014). Transposable element islands facilitate adaptation to novel environments in an invasive species. Nat Commun.

[51] Yi S, Streelman JT (2005). Genome size is negatively correlated with effective population size in ray-finned fish. Trends Genet.

[52] Howe K, Clark MD, Torroja CF, Torrance J, Berthelot C, Muffato M, Collins JE, Humphray S, McLaren K, Matthews L (2013). The zebrafish reference genome sequence and its relationship to the human genome. Nature.

[53] Lynch M, Conery JS (2003). The origins of genome complexity. Science.

[54] Liu Z, Liu S, Yao J, Bao L, Zhang J, Li Y, Jiang C, Sun L, Wang R, Zhang Y (2016). The channel catfish genome sequence provides insights into the evolution of scale formation in teleost. Nat Commun.

[55] Sullivan JP, Lundberg JG, Hardman M (2006). A phylogenetic analysis of the major groups of catfishes (Teleostei: Siluriformes) using rag1 and rag2 nuclear gene sequences [J]. Mol Phylogenet Evol.

[56] Ackerly DD, Reich PB (1999). Convergence and correlations among leaf size and function in seed plants: a comparative test using independent contrasts. Am J Bot.

[57] McPhail J (1993). Ecology and evolution of sympatric sticklebacks (*Gasterosteus*): origin of the species pairs. Can J Zool.

[58] McPhail J (1994). Speciation and the evolution of reproductive isolation in the sticklebacks (*Gasterosteus*) of south-western British Columbia. The evolutionary biology of the threespine stickleback.

[59] Jones FC, Grabherr MG, Chan YF, Russell P, Mauceli E, Johnson J, Swofford R, Pirun M, Zody MC, White S (2012). The genomic basis of adaptive evolution in threespine sticklebacks. Nature.

[60] Parry G (1966). Osmotic adaptation in fishes. Biol Rev.

[61] Yancey PH, Clark ME, Hand SC, Bowlus RD, Somero GN (1982). Living with water stress: evolution of osmolyte systems. Science.

[62] Canceill D, Ehrlich SD (1996). Copy-choice recombination mediated by DNA polymerase III holoenzyme from *Escherichia coli*. Proc Natl Acad Sci.

[63] Fraser BA, Künstner A, Reznick DN, Dreyer C, Weigel D (2015). Population genomics of natural and experimental populations of guppies (Poecilia Reticulata). Mol Ecol.

[64] Schartl M, Walter RB, Shen Y, Garcia T, Catchen J, Amores A, Braasch I, Chalopin D, Volff J-N, Lesch K-P (2013). The genome of the platyfish, *Xiphophorus maculatus*, provides insights into evolutionary adaptation and several complex traits. Nat Genet.

[65] Kasahara M, Naruse K, Sasaki S, Nakatani Y, Qu W, Ahsan B, Yamada T, Nagayasu Y, Doi K, Kasai Y (2007). The medaka draft genome and insights into vertebrate genome evolution. Nature.

[66] Brawand D, Wagner CE, Li YI, Malinsky M, Keller I, Fan S, Simakov O, Ng AY, Lim ZW, Bezault E (2014). The genomic substrate for adaptive radiation in African cichlid fish. Nature.

[67] Conte MA, Kocher TD (2015). An improved genome reference for the African cichlid, Metriaclima Zebra. BMC Genomics.

[68] McGaugh SE, Gross JB, Aken B, Blin M, Borowsky R, Chalopin D, Hinaux H, Jeffery WR, Keene A, Ma L (2014). The cavefish genome reveals candidate genes for eye loss. Nat Commun.

[69] Barrio AM, Lamichhaney S, Fan G, Rafati N, Pettersson M, Zhang H, Dainat J, Ekman D, Höppner M, Jern P (2016). The genetic basis for ecological adaptation of the Atlantic herring revealed by genome sequencing. elife.

[70] Shin SC, Ahn DH, Kim SJ, Pyo CW, Lee H, Kim M-K, Lee J, Lee JE, Detrich HW, Postlethwait JH (2014). The genome sequence of the Antarctic bullhead notothen reveals evolutionary adaptations to a cold environment. Genome Biol.

[71] Tine M, Kuhl H, Gagnaire P-A, Louro B, Desmarais E, Martins RS, Hecht J, Knaust F, Belkhir K, Klages S (2014). European sea bass genome and its variation provide insights into adaptation to euryhalinity and speciation. Nat Commun.

[72] Smolka M, Rescheneder P, Schatz MC, von Haeseler A, Sedlazeck FJ (2015). Teaser: individualized benchmarking and optimization of read mapping results for NGS data. Genome Biol.

[73] AlMomin S, Kumar V, Al-Amad S, Al-Hussaini M, Dashti T, Al-Enezi K, Akbar A (2015). Draft genome sequence of the silver pomfret fish, Pampus Argenteus. Genome.

[74] Nakamura Y, Mori K, Saitoh K, Oshima K, Mekuchi M, Sugaya T, Shigenobu Y, Ojima N, Muta S, Fujiwara A (2013). Evolutionary changes of multiple visual pigment genes in the complete genome of Pacific bluefin tuna. Proc Natl Acad Sci.

[75] Wu C, Zhang D, Kan M, Lv Z, Zhu A, Su Y, Zhou D, Zhang J, Zhang Z, Xu M (2014). The draft genome of the large yellow croaker reveals well-developed innate immunity. Nat Commun.

[76] Xu T, Xu G, Che R, Wang R, Wang Y, Li J, Wang S, Shu C, Sun Y, Liu T (2016). The genome of the miiuy croaker reveals well-developed innate immune and sensory systems. Sci Rep.

[77] Chen S, Zhang G, Shao C, Huang Q, Liu G, Zhang P, Song W, An N, Chalopin D, Volff J-N (2014). Whole-genome sequence of a flatfish provides insights into ZW sex chromosome evolution and adaptation to a benthic lifestyle. Nat Genet.

[78] Aparicio S, Chapman J, Stupka E, Putnam N, Chia JM, Dehal P, Christoffels A, Rash S, Hoon S, Smit A (2002). Whole-genome shotgun assembly and analysis of the genome of Fugu Rubripes. Science.

[79] Gao Y, Gao Q, Zhang H, Wang L, Zhang F, Yang C, Song L (2014). Draft sequencing and analysis of the genome of pufferfish Takifugu Flavidus. DNA Res.

[80] Lien S, Koop BF, Sandve SR, Miller JR, Kent MP, Nome T, Hvidsten TR, Leong JS, Minkley DR, Zimin A (2016). The Atlantic salmon genome provides insights into rediploidization. Nature.

[81] Rondeau EB, Minkley DR, Leong JS, Messmer AM, Jantzen JR, von Schalburg KR, Lemon C, Bird NH, Koop BF (2014). The genome and linkage map of the northern pike (*Esox lucius*): conserved synteny revealed between the salmonid sister group and the Neoteleostei. PLoS One.

[82] Burns FR, Cogburn AL, Ankley GT, Villeneuve DL, Waits E, Chang YJ, Llaca V, Deschamps SD, Jackson RE, Hoke RA (2016). Sequencing and de novo draft assemblies of a fathead minnow (Pimephales Promelas) reference genome. Environ Toxicol Chem.

[83] Yang J, Chen X, Bai J, Fang D, Qiu Y, Jiang W, Yuan H, Bian C, Lu J, He S (2016). The Sinocyclocheilus cavefish genome provides insights into cave adaptation. BMC Biol.

[84] Star B, Nederbragt AJ, Jentoft S, Grimholt U, Malmstrøm M, Gregers TF, Rounge TB, Paulsen J, Solbakken MH, Sharma A (2011). The genome sequence of Atlantic cod reveals a unique immune system. Nature.

[85] Braasch I, Gehrke AR, Smith JJ, Kawasaki K, Manousaki T, Pasquier J, Amores A, Desvignes T, Batzel P, Catchen J (2016). The spotted gar genome illuminates vertebrate evolution and facilitates human-teleost comparisons. Nat Genet.

[86] Amemiya CT, Alföldi J, Lee AP, Fan S, Philippe H, MacCallum I, Braasch I, Manousaki T, Schneider I, Rohner N (2013). The African coelacanth genome provides insights into tetrapod evolution. Nature.

[87] Read TD, Petit RA, Joseph SJ, Alam MT, Weil R, Ahmad M, Bhimani R, Vuong JS, Haase CP, Webb H (2015). Draft sequencing and assembly of the genome of the world’s largest fish, the whale shark: *Rhincodon typus* smith 1828. Peer J Pre Prints.

[88] Venkatesh B, Lee AP, Ravi V, Maurya AK, Lian MM, Swann JB, Ohta Y, Flajnik MF, Sutoh Y, Kasahara M (2014). Elephant shark genome provides unique insights into gnathostome evolution. Nature.

[89] Smith JJ, Kuraku S, Holt C, Sauka-Spengler T, Jiang N, Campbell MS, Yandell MD, Manousaki T, Meyer A, Bloom OE (2013). Sequencing of the sea lamprey (*Petromyzon marinus*) genome provides insights into vertebrate evolution. Nat Genet.

[90] Bao Z, Eddy SR (2002). Automated de novo identification of repeat sequence families in sequenced genomes. Genome Res.

[91] Price AL, Jones NC, Pevzner PA (2005). De novo identification of repeat families in large genomes. Bioinformatics.

[92] Wheeler TJ, Clements J, Eddy SR, Hubley R, Jones TA, Jurka J, Smit AF, Finn RD (2013). Dfam: a database of repetitive DNA based on profile hidden Markov models. Nucleic Acids Res.

[93] Bao W, Kojima KK, Kohany O (2015). Repbase update, a database of repetitive elements in eukaryotic genomes. Mob DNA.

[94] Castresana J (2001). Cytochrome b phylogeny and the taxonomy of great apes and mammals. Mol Biol Evol.

[95] Katoh K, Standley DM (2013). MAFFT multiple sequence alignment software version 7: improvements in performance and usability. Mol Biol Evol.

[96] Darriba D, Taboada GL, Doallo R, Posada D (2011). ProtTest 3: fast selection of best-fit models of protein evolution. Bioinformatics.

[97] Kumar S, Stecher G, Tamura K (2016). MEGA7: molecular evolutionary genetics analysis version 7.0 for bigger datasets. Mol Biol Evol.

[98] Chinwalla AT, Cook LL, Delehaunty KD, Fewell GA, Fulton LA, Fulton RS, Graves TA, Hillier LW, Mardis ER, McPherson JD (2002). Initial sequencing and comparative analysis of the mouse genome. Nature.

[99] R Core Team. R: A language and environment for statistical computing. R foundation for statistical computing, Vienna, Austria. 2003. http://www.R-project.org/.

[100] Fofonoff NP (1985). Physical properties of seawater: a new salinity scale and equation of state for seawater. J Geophys Res-Oceans.

[101] Paradis E, Claude J, Strimmer K (2004). APE: analyses of phylogenetics and evolution in R language[J]. Bioinformatics.

[102] Deng W, Wang Y, Liu Z, Cheng H, Xue Y (2014). HemI: a toolkit for illustrating heatmaps. PLoS One.

