# Supplementary material for: Comparative genome analysis of 52 fish species suggests differential associations of repetitive elements with their living aquatic environments
Source: BMC Genomics. 2018 Feb 13;19:141. doi: 10.1186/s12864-018-4516-1 (PMC5811955; doi:10.1186/s12864-018-4516-1)
Supplement: Supplementary file 1 — Fish genomes used for analysis. (DOCX 33 kb) [file 12864_2018_4516_MOESM1_ESM.docx]

**Supplemental Table 1.** Fish genomes used for analysis, with their habitats indicated by color

| Order | Species | Habitats | Genome Size (MB) | Reference OR Accession |
| --- | --- | --- | --- | --- |
| Cyprinodontiformes | *Poecilia Formosa* | Freshwater | 748.9 | GCA_000485575.1 |
| Cyprinodontiformes | *Poecilia Mexicana* | Freshwater | 801.7 | GCA_001443325.1 |
| Cyprinodontiformes | *Poecilia latipinna* | Freshwater | 815.1 | GCA_001443285.1 |
| Cyprinodontiformes | *Poecilia reticulata* | Freshwater | 731.6 | GCA_000633615.2 [1] |
| Cyprinodontiformes | *Xiphophorus couchianus* | Freshwater | 708.4 | GCA_001444195.1 [2] |
| Cyprinodontiformes | *Xiphophorus hellerii* | Freshwater | 733.8 | GCA_001443345.1 [2] |
| Cyprinodontiformes | *Xiphophorus maculatus* | Freshwater | 729.7 | GCA_000241075.1 [2] |
| Cyprinodontiformes | *Fundulus heteroclitus* | Freshwater | 1021.9 | GCA_000826765.1 |
| Cyprinodontiformes | *Cyprinodon variegatus* | Freshwater | 1035.2 | GCA_000732505.1 |
| Cyprinodontiformes | *Cyprinodon nevadensis* | Freshwater | 1011.9 | GCA_000776015.1 |
| Beloniformes | *Oryzias latipes* | Freshwater | 869.8 | MEDAKA1 [3] |
| Perciformes | *Amphilophus citrinellus* | Freshwater | 844.9 | GCA_000751415.1 |
| Perciformes | *Neolamprologus brichardi* | Freshwater | 847.9 | GCA_000239395.1 [4] |
| Perciformes | *Pundamilia nyererei* | Freshwater | 830.1 | GCA_000239375.1 [4] |
| Perciformes | *Haplochromis burtoni* | Freshwater | 831.4 | GCA_000239415.1 [4] |
| Perciformes | *Oreochromis niloticus* | Freshwater | 1009.9 | GCA_000188235.1 [4] |
| Perciformes | *Maylandia zebra* | Freshwater | 859.8 | GCA_000238955.3 [5] |
| Siluriformes | *Ictalurus punctatus* | Freshwater | 942.2 | [6]. |
| Characiformes | *Astyanax mexicanus* | Freshwater | 1191.2 | GCA_000372685.1 [7] |
| Clupeiformes | *Clupea harengus* | Marine | 807.7 | GCA_000966335.1 [8] |
| Perciformes | *Notothenia coriiceps* | Marine | 636.6 | GCA_000735185.1 [9] |
| Perciformes | *Dicentrarchus labrax* | Marine | 675.9 | GCA_000689215.1 [10] |
| Gasterosteiformes | *Gasterosteus aculeatus* | Marine | 446.6 | BROAD S1 [11] |
| Scorpaeniformes | *Cottus rhenanus* | Freshwater | 563.6 | GCA_001455555.1 [12] |
| Scorpaeniformes | *Sebastes nigrocinctus* | Marine | 746.0 | GCA_000475235.1 |
| Scorpaeniformes | *Sebastes rubrivinctus* | Marine | 756.3 | GCA_000475215.1 |
| Perciformes | *Pampus argenteus* | Marine | 350.4 | GCA_000697985.1 [13] |
| Perciformes | *Thunnus orientalis* | Marine | 684.5 | GCA_000418415.1 [14] |
| Perciformes | *Larimichthys crocea* | Marine | 678.9 | GCA_000742935.1 [15] |
| Perciformes | *Miichthys miiuy* | Marine | 619.3 | GCA_001593715.1 [16] |
| Pleuronectiformes | *Cynoglossus semilaevis* | Marine | 470.2 | GCA_000523025.1 [17] |
| Tetraodontiformes | *Tetraodon nigroviridis* | Freshwater | 342.4 | TETRAODON 8.0 [18] |
| Tetraodontiformes | *Takifugu rubripes* | Marine | 391.5 | FUGU 4.0 [19] |
| Tetraodontiformes | *Takifugu flavidus* | Marine | 378.0 | GCA_000400755.1 [20] |
| Salmoniformes | *Salmo salar* | Diadromous | 2966.9 | GCA_000233375.4 [21] |
| Esociformes | *Esox lucius* | Freshwater | 904.5 | GCA_000721915.2 [22] |
| Cypriniformes | *Pimephales promelas* | Freshwater | 1219.3 | GCA_000700825.1 [23] |
| Cypriniformes | *Sinocyclocheilus rhinocerous* | Freshwater | 1655.8 | GCA_001515625.1 [24] |
| Cypriniformes | *Sinocyclocheilus grahami* | Freshwater | 1750.3 | GCA_001515645.1 [24] |
| Cypriniformes | *Sinocyclocheilus anshuiensis* | Freshwater | 1632.7 | GCA_001515605.1 [24] |
| Cypriniformes | *Cyprinus carpio* | Freshwater | 1713.7 | GCA_000951615.1 [25] |
| Cypriniformes | *Danio rerio* | Freshwater | 1679.2 | GRCz10 |
| Gadiformes | *Gadus morhua* | Marine | 824.3 | GCA_000231765.1 [26] |
| Anguilliformes | *Anguilla anguilla* | Diadromous | 1018.7 | GCA_000695075.1 |
| Anguilliformes | *Anguilla rostrata* | Diadromous | 1413.0 | GCA_001606085.1 |
| Lepisosteiformes | *Lepisosteus oculatus* | Freshwater | 945.9 | GCA_000242695.1 [27] |
| Coelacanthiformes | *Latimeria chalumnae* | Marine | 2860.6 | GCA_000225785.1 [28] |
| Rajiformes | *Leucoraja erinacea* | Marine | 1555.5 | GCA_000238235.1 |
| Orectolobiformes | *Rhincodon typus* | Marine | 2931.6 | GCA_001642345.1 [29] |
| Chimaeriformes | *Callorhinchus milii* | Marine | 974.5 | GCA_000165045.2 [30] |
| Petromyzontiforme | *Lethenteron camtschaticum* | Freshwater | 1030.7 | GCA_000466285.1 |
| Petromyzontiformes | *Petromyzon marinus* | Freshwater | 885.5 | Pmarinus_7.0 [31] |

**Supplementary Reference**

1.Fraser BA, Künstner A, Reznick DN, Dreyer C, Weigel D. Population genomics of natural and experimental populations of guppies (*Poecilia reticulata*). Mol Ecol. 2015;24:389-408.

2. Schartl M, Walter RB, Shen Y, Garcia T, Catchen J, Amores A, Braasch I, Chalopin D, Volff J-N, Lesch K-P. The genome of the platyfish, *Xiphophorus maculatus*, provides insights into evolutionary adaptation and several complex traits. Nat Genet. 2013;45:567-572.

3. Kasahara M, Naruse K, Sasaki S, Nakatani Y, Qu W, Ahsan B, Yamada T, Nagayasu Y, Doi K, Kasai Y. The medaka draft genome and insights into vertebrate genome evolution. Nature. 2007;447:714-9.

4. Brawand D, Wagner CE, Li YI, Malinsky M, Keller I, Fan S, Simakov O, Ng AY, Lim ZW, Bezault E. The genomic substrate for adaptive radiation in African cichlid fish. Nature. 2014;513:375-81.

5. Conte MA, Kocher TD. An improved genome reference for the African cichlid, *Metriaclima zebra*. BMC Genomics. 2015;16:724.

6. Liu Z, Liu S, Yao J, Bao L, Zhang J, Li Y, Jiang C, Sun L, Wang R, Zhang Y. The channel catfish genome sequence provides insights into the evolution of scale formation in teleosts. Nat Commun. 2016;7:11757.

7. McGaugh SE, Gross JB, Aken B, Blin M, Borowsky R, Chalopin D, Hinaux H, Jeffery WR, Keene A, Ma L. The cavefish genome reveals candidate genes for eye loss. Nat Commun. 2014;5:5307.

8. Barrio AM, Lamichhaney S, Fan G, Rafati N, Pettersson M, Zhang H, Dainat J, Ekman D, Höppner M, Jern P. The genetic basis for ecological adaptation of the Atlantic herring revealed by genome sequencing. eLife. 2016;5:e12081.

9. Shin SC, Ahn DH, Kim SJ, Pyo CW, Lee H, Kim M-K, Lee J, Lee JE, Detrich HW, Postlethwait JH. The genome sequence of the Antarctic bullhead notothen reveals evolutionary adaptations to a cold environment. Genome Biol. 2014;15:468.

10. Tine M, Kuhl H, Gagnaire P-A, Louro B, Desmarais E, Martins RS, Hecht J, Knaust F, Belkhir K, Klages S. European sea bass genome and its variation provide insights into adaptation to euryhalinity and speciation. Nat Commun. 2014;5:5770.

11.Jones FC, Grabherr MG, Chan YF, Russell P, Mauceli E, Johnson J, Swofford R, Pirun M, Zody MC, White S: The genomic basis of adaptive evolution in threespine sticklebacks. Nature. 2012;484(7392):55-61.

12. Smolka M, Rescheneder P, Schatz MC, von Haeseler A, Sedlazeck FJ. Teaser: Individualized benchmarking and optimization of read mapping results for NGS data. Genome Biol. 2015;16:235.

13. AlMomin S, Kumar V, Al-Amad S, Al-Hussaini M, Dashti T, Al-Enezi K, Akbar A. Draft genome sequence of the silver pomfret fish, Pampus argenteus. Genome. 2015;59:51-8.

14. Nakamura Y, Mori K, Saitoh K, Oshima K, Mekuchi M, Sugaya T, Shigenobu Y, Ojima N, Muta S, Fujiwara A. Evolutionary changes of multiple visual pigment genes in the complete genome of Pacific bluefin tuna. Proc Natl Acad Sci. 2013;110:11061-6.

15. Wu C, Zhang D, Kan M, Lv Z, Zhu A, Su Y, Zhou D, Zhang J, Zhang Z, Xu M. The draft genome of the large yellow croaker reveals well-developed innate immunity. Nat Commun. 2014;5:5227.

16. Xu T, Xu G, Che R, Wang R, Wang Y, Li J, Wang S, Shu C, Sun Y, Liu T. The genome of the miiuy croaker reveals well-developed innate immune and sensory systems. Sci Rep. 2016;6:21902.

17. Chen S, Zhang G, Shao C, Huang Q, Liu G, Zhang P, Song W, An N, Chalopin D, Volff J-N. Whole-genome sequence of a flatfish provides insights into ZW sex chromosome evolution and adaptation to a benthic lifestyle. Nat Genet. 2014;46:253-60.

18. Jaillon O, Aury J-M, Brunet F, Petit J-L, Stange-Thomann N, Mauceli E, Bouneau L, Fischer C, Ozouf-Costaz C, Bernot A. Genome duplication in the teleost fish *Tetraodon nigroviridis* reveals the early vertebrate proto-karyotype. Nature. 2004;431:946-57.

19.Aparicio S, Chapman J, Stupka E, Putnam N, Chia J-m, Dehal P, Christoffels A, Rash S, Hoon S, Smit A. Whole-genome shotgun assembly and analysis of the genome of *Fugu rubripes*. Science. 2002;297:1301-10.

20. Gao Y, Gao Q, Zhang H, Wang L, Zhang F, Yang C, Song L. Draft Sequencing and Analysis of the Genome of Pufferfish *Takifugu flavidus*. Dna Res. 2014;21:627-37.

21. Lien S, Koop BF, Sandve SR, Miller JR, Kent MP, Nome T, Hvidsten TR, Leong JS, Minkley DR, Zimin A. The Atlantic salmon genome provides insights into rediploidization. Nature. 2016;533:200-5.

22. Rondeau EB, Minkley DR, Leong JS, Messmer AM, Jantzen JR, von Schalburg KR, Lemon C, Bird NH, Koop BF. The genome and linkage map of the northern pike (*Esox lucius*): conserved synteny revealed between the salmonid sister group and the Neoteleostei. PloS one. 2014;9:e102089.

23. Burns FR, Cogburn AL, Ankley GT, Villeneuve DL, Waits E, Chang YJ, Llaca V, Deschamps SD, Jackson RE, Hoke RA. Sequencing and de novo draft assemblies of a fathead minnow (*Pimephales promelas*) reference genome. Environ Toxicol Chem. 2016;35:212-7.

24.Yang J, Chen X, Bai J, Fang D, Qiu Y, Jiang W, Yuan H, Bian C, Lu J, He S. The Sinocyclocheilus cavefish genome provides insights into cave adaptation. BMC Biol. 2016;14:1.

25. Xu P, Zhang X, Wang X, Li J, Liu G, Kuang Y, Xu J, Zheng X, Ren L, Wang G. Genome sequence and genetic diversity of the common carp, *Cyprinus carpio*. Nat Genet. 2014;46:1212-9.

26. Star B, Nederbragt AJ, Jentoft S, Grimholt U, Malmstrøm M, Gregers TF, Rounge TB, Paulsen J, Solbakken MH, Sharma A. The genome sequence of Atlantic cod reveals a unique immune system. Nature. 2011;477:207-10.

27. Braasch I, Gehrke AR, Smith JJ, Kawasaki K, Manousaki T, Pasquier J, Amores A, Desvignes T, Batzel P, Catchen J. The spotted gar genome illuminates vertebrate evolution and facilitates human-teleost comparisons. Nat Genet. 2016;48:427-37.

28. Amemiya CT, Alföldi J, Lee AP, Fan S, Philippe H, MacCallum I, Braasch I, Manousaki T, Schneider I, Rohner N. The African coelacanth genome provides insights into tetrapod evolution. Nature. 2013;496:311-6.

29. Read TD, Petit III RA, Joseph SJ, Alam MT, Weil R, Ahmad M, Bhimani R, Vuong JS, Haase CP, Webb H. Draft sequencing and assembly of the genome of the world’s largest fish, the whale shark: *Rhincodon typus* Smith 1828. PeerJ PrePrints. 2015;14:837v1.

30. Venkatesh B, Lee AP, Ravi V, Maurya AK, Lian MM, Swann JB, Ohta Y, Flajnik MF, Sutoh Y, Kasahara M. Elephant shark genome provides unique insights into gnathostome evolution. Nature. 2014;505:174-9.

31. Smith JJ, Kuraku S, Holt C, Sauka-Spengler T, Jiang N, Campbell MS, Yandell MD, Manousaki T, Meyer A, Bloom OE. Sequencing of the sea lamprey (*Petromyzon marinus*) genome provides insights into vertebrate evolution. Nat Genet. 2013;45:415-21.
